# Sugarcane Functional Genomics: Gene Discovery for Agronomic Trait Development

**DOI:** 10.1155/2008/458732

**Published:** 2007-12-16

**Authors:** M. Menossi, M. C. Silva-Filho, M. Vincentz, M.-A. Van-Sluys, G. M. Souza

**Affiliations:** ^1^Departmento de Genetica e Evolução IB-Unicamp, Centro de Biologia Molecular e Engenharia Genética, Universidade Estadual de Campinas, C.P. 6010, CEP 13083-970 Campinas, SP, Brazil; ^2^Departamento de Genética, Escola Superior de Agricultura Luiz de Queiroz, Universidade de São Paulo, Av. Pádua Dias, 11, C.P. 83, 13400-970 Piracicaba, SP, Brazil; ^3^Departamento de Botânica, Instituto de Biociências, Universidade de São Paulo, Rua do Matão 277, 05508-090 São Paulo, SP, Brazil; ^4^Departamento de Bioquímica, Instituto de Química, Universidade de São Paulo, Av. Prof. Lineu Prestes 748, 05508-900 São Paulo, SP, Brazil

## Abstract

Sugarcane is a highly productive crop used for centuries as the main source of sugar and recently to produce ethanol, a renewable bio-fuel energy source. There is increased interest in this crop due to the impending need to decrease fossil fuel usage. Sugarcane has a highly polyploid genome. Expressed sequence tag (EST) sequencing has significantly contributed to gene discovery and expression studies used to associate function with sugarcane genes. A significant amount of data exists on regulatory events controlling responses to herbivory, drought, and phosphate deficiency, which cause important constraints on yield and on endophytic bacteria, which are highly beneficial. The means to reduce drought, phosphate deficiency, and herbivory by the sugarcane borer have a negative impact on the environment. Improved tolerance for these constraints is being sought. Sugarcane's ability to accumulate sucrose up to 16% of its culm dry weight is a challenge for genetic manipulation. Genome-based technology such as cDNA microarray data indicates genes associated with sugar content that may be used to develop new varieties improved for sucrose content or for traits that restrict the expansion of the cultivated land. The genes can also be used as molecular markers of agronomic traits in traditional breeding programs.

## 1. SUGARCANE: A HIGHLY SUCCESSFUL CROP WITH A CHALLENGING GENOME

Sugarcane is an important tropical crop and has served as a source of sugar for hundreds of years. With an originally soft, watery culm sugarcane acquired through human selection a distinctive feature of partitioning carbon into sucrose in the stem. The striking ability of accumulating levels of sucrose that can reach around 0.7 M in mature internodes [1] is an almost unique feature in cultivated plants.

Sugarcane is cultivated in more than 20 million hectares in tropical and subtropical regions of the world, producing up to 1.3 billion metric tons of crushable stems. It is generally used to produce sugar, accounting for almost two thirds of the world’s production and has recently gained increased attention because ethanol derived from cane sugar represents an important renewable biofuel source, which could turn it into a global commodity and important energy source. Sugarcane bagasse (the major waste product generated by sugar mills after extraction of the sucrose from cane juice) is largely used for energy cogeneration at the mill or for the production of animal feed increasing the overall efficiency of the crop system. Recently, there has been increased interest in using bagasse for processes such as paper production, as a dietary fiber in bread, as a wood substitute in the production of wood composite, and in the synthesis of carbon fibres [2–6]. It is expected that enzymatic and hydrolytic processes that allow the bagasse carbon units from cellulose and hemicellulose to be fermented, will soon be scaled up for ethanol production, turning sugarcane into an efficient crop for energy production.

Commercial sugarcane relies on vegetative propagation through stem cuttings to generate a new clonal plant, resulting from lateral bud growth, and subsequently stools, with a large number of tillers. In 12 months the plant will reach 4-5 meters, with extractable culms measuring 2-3 meters and a sugar content of 13–16%. After harvest, underground buds will sprout starting a new crop season. In most situations 4–6 harvests are possible before the field is renewed. After each harvest, leaves and plant toppings removed from the stems are left in the fields allowing for nutrient recycling, soil protection and growth without crop rotation.

Sugarcane belongs to the genus *Saccharum* L composed of hybrids [7, 8] derived from *Saccharum officinarum* (Noble clones), *S. sinense* (Chinese clones), *S. barberi* (North Indian clones), and *S. spontaneum* [9]. The hybrids are highly polyploid and aneuploid and on average contain 100–120 chromosomes with an estimated somatic cell size of 10,000 Mbp [10]. The number of chromosomes can vary in commercial cultivars. The basic genome size ranges from 760 to 926 Mbp, which is twice the size of the rice genome (389 Mbp) and similar to sorghum's (760 Mbp) [11]. Even in the face of the economic importance, it represents to many countries, the complexity of the sugarcane genome inhibited large efforts and investments in the development of biotechnology and genetic tools for this crop. Cultivar improvement has been achieved over the years using traditional breeding, which can take up to 15 years of selections. Nevertheless sugarcane transgenics are still lagging behind. Herbicide-, herbivory-, and viral-resistant transgenic plants have been reported but so far there has been no commercial release. This is probably due to intellectual property and regulatory issues, but may also be related to the fact that for complex traits, such as sucrose content, the genes to be used have not yet been proved ideal for improving agronomic performance. Gene discovery and identification is essential for breeding programs, either for transgenic plant development or for marker-assisted breeding.

The complete genome sequence of a sugarcane cultivar is not yet available. Significant progress has been noted recently with the development of tools such as expressed sequence tags (ESTs). Large collections have become available to explore the large polyploid sugarcane genome and consequently renewed the interest in sugarcane genetics [12–14]. This review will focus on describing EST development and subsequent progress that led to the identification of genes associated with agronomic traits of interest in sugarcane. It will also highlight some of the possible functions of genes associated with sucrose content, including biotic and abiotic stress and the role that phytohormones may play in the adaptive responses of this plant.

## 2. EST PROFILING FOR GENE DISCOVERY

ESTs represent tags of the expressed portion of a genome and therefore potentially identify genes encoding proteins, natural antisense transcripts [15–18], miRNA, transacting siRNA precursors [19, 20], and more generally noncoding RNA [21]. The information carried by an EST collection is a significant starting point to determine an organism's genome content but more pragmatically, when considering important crops, it can directly point to genes which may contribute to agronomical trait development (e.g., tolerance to abiotic and biotic stresses, mineral nutrition, and sugar content amongst others).

Several sugarcane ESTs collections have been developed [22–28]. The publicly available sugarcane ESTs were assembled into tentative consensus sequences (virtual transcripts), singletons, and mature transcripts, referred to as the Sugarcane Gene Index (SGI; http://compbio.dfci.harvard.edu/tgi/cgi-bin/tgi/gimain.pl?gudb=s_officinarum). The Brazilian sugarcane EST project collection (SUCEST, http://sucest-fun.org, [26]) generated 237,954 ESTs, which were organized into 43,141 putative unique sugarcane transcripts (26,803 contigs and 16,338 singletons) referred to as sugarcane assembled sequences (SASs). An internal redundancy analysis suggested that this collection of SASs represented 33,000 sugarcane genes [13, 26, 29] but this estimation was likely to have been an overestimation, since a two-fold redundancy among SASs that presented significant similarity with *Arabidopsis* proteins (60% of the SASs) was detected (M. Vincentz, unpublished data). A detailed organization of sugarcane genes into functional categories (i.e., signal transduction components, regulation of gene expression, development, biotic and abiotic stresses, transposable elements, metabolism, etc., [26]) was completed and represents the basis to develop functional genomic approaches.

The contribution of this large set of SASs to our understanding of the processes underlying angiosperm evolution was also of significance. A comparison of the SASs with the DNA and protein sequences from other angiosperms confirmed that lineage-specific gene loss, high evolutionary rate of specific sequences, and exon shuffling were important processes involved in the divergence among angiosperms [30]. Of particular interest are the monocot-specific sequences that evolve at high rates and are found in members of conserved angiosperm gene families, because they may lead to functional diversification and may therefore be related to the differentiation of specific lineages. Interestingly, two SASs (SCEZSD2038A10.g and SCSFRT2070F09.g), only detected in sugarcane, sorghum and maize, point to the existence of recent innovations in the Andropogoneae tribe (M. Vincentz, unpublished) and raise the question of what kind of adaptive traits are associated with these sequences.

Finally, it is important to note the contribution that the EST collections have made to our understanding of the sugarcane genome structure, number of alleles and the complex relationship of specific alleles and allele dosage to phenotypes. Single sequence repeats (SSRs) and single nucleotide polymorphisms (SNPs) have been annotated in a number of genotypes [31, 32]; and with the advent of pyrosequencing, their identification has been increasingly adding to our knowledge of large genomes [33]. A comprehensive functional map of the sugarcane genome has recently been described with an enhanced resolution, creating the means for developing ``perfect markers'' associated with key QTL [34].

## 3. GENE EXPRESSION BLUEPRINT OF SUGARCANE TISSUES

The availability of ESTs allows for large-scale gene expression analysis using a variety of tools. Several studies have reported an in silicoanalysis of transcript enrichment when different cDNA libraries were compared [26, 35]. Of the 43,141 SUCEST SASs, 1234 were considered to be tissue-enriched. The maximum number of ESTs in a tissue-enriched SAS (i.e., with higher transcript amounts in one or more tissues) was found to be in prolamin, which contained 360 ESTs. Developing seeds contained 1902 specific ESTs (33% of the total), with almost half of these (919) encoding prolamins, the major seed storage protein found in cereals. These ESTs included six putative new genes with a high level of expression in seeds (up to 32 ESTs/SAS). The most frequent protein domains found in tissue-enriched SASs were the protein kinase domain, followed by the trypsin-amylase inhibitor, seed storage protein, and lipid transfer protein domains. Overall, 13 transcription factor families were found to be specific for flowers, five for roots, three for *Herbaspirillum*-inoculated plantlets, and two for developing seeds and other tissues.

Following sequence identification, a functional genomics project, the SUCEST-FUN Project (http:sucest-fun.org), was implemented to associate putative roles with the sugarcane genes. cDNA microarrays containing sugarcane ESTs were used to determine temporal and spatial gene expression. Determining the distribution of gene transcripts in sugarcane tissues has helped define tissue-specific activities and ubiquitous genes, and point out genes in which promoter sequences could be searched for. This is of particular interest if one is interested in directing the expression of transgenes to particular plant tissues to avoid pleiotropic effects. Individual gene expression variation was investigated using cDNA microarrays containing 1280 distinct elements, on plants grown in the field. Transcript abundance in six plant organs (flowers, roots, leaves, lateral buds, 1st (immature), and 4th (mature) internodes) was analyzed [36], resulting in the identification of 217 genes with a tissue-enriched expression patterns, while 153 genes showed highly similar expression levels in all the tissues analyzed. A virtual profile matrix was constructed where tissue expression was compared amongst 24 tissue samples. Amongst the tissue-enriched genes,a caffeic acid 3-*O*-methyltransferase (COMT) gene expressed primarily in the mature internode was identified. This enzyme is involved in lignin biosynthesis and, in association with other enzymes like the CCOMT (caffeoyl CoA 3-*O*-methyltransferase), keeps the cell lignin content and composition in check. The identification of this culm-enriched enzyme may lead to improved sugarcane varieties with an altered lignin content: a trait highly valuable for the paper industry and for those interested in increasing hydrolysis of the sugarcane bagasse for fermentation purposes. The tissue specificity data was also evaluated against data from plants submitted to biotic and abiotic stresses, which can shed light on the putative roles of newly identified genes, such as genes for which no similarity has been found with genes in the public databases [37].

Active transcription of transposable elements (TEs) was also detected in the SUCEST database [38] and enabled the identification of a previously unknown set of genetic mobile elements in sugarcane. Further studies confirmed the expression profile of 68 individual TE clones [39]. Four actively dividing tissues were examined (callus, apical meristem, leaf roll, and flower), and callus was determined to be the tissue expressing the most diverse group of TEs. Both transposons and retrotransposons are expressed, which suggest that some of these mobile elements may have an important role in genome metabolism, as previously described for other elements in several biological systems [40, 41]. Further analysis of these transcribed TEs revealed that some of the families were constituted by both bona fide transposable elements and domesticated variants that had been captured by the plant genome to perform a yet unknown function [42]. Mutator-like elements were the most expressed transposons in sugarcane, and four groups were identified that showed similarity with the MURA transposase protein [43], of which two represented domesticated elements related to the Mustang-like genes described in rice [42, 44]. Amongst the retrotransposons, Hopscotch-like sequences, the most prevalent in the SUCEST database, showed a highly diverse expression profile. Retrotransposons carry their promoter region along the length of their transcribed mRNA and GUS-fusion expression analyses for three out of four TEs, this is being confirmed in transient assays [44], leading to the possibility of using these sequences as promoters for the expression of genes of interest in sugarcane.

## 4. INSIGHTS IN THE SUGARCANE RESPONSES TO BIOTIC STRESS

Plants are constantly challenged by a wide array of biotic stresses, such as herbivorous insects, nematodes, and by fungal, bacterial, and viral infestations. Phytohormones largely mediate plant responses to attacks by triggering conserved defence mechanisms, each with an intricate signalling pathway leading to plant protection. Cross-talk signalling pathways leading to plant defence have been reported, with synergistic and antagonistic outcomes [45]. Specific and general responses are mediated by distinct signals, mainly jasmonic acid, ethylene, and salicylic acid. It has been shown that both the ethylene and jasmonic acid signalling pathways act synergistically in plant defence. For example, ethylene synthesis increases the response to severaltypes of biotic challenges (e.g., bacteria, fungi [46], and insects [47]). In sugarcane, a putative ethylene receptor and two putative transcription factors, which are members of the ethylene signalling pathway, have been shown to be regulated during the association with nitrogen-fixing endophytic bacteria [48]. In addition, other signals such as green leaf volatiles (GLVs) may be involved in the orchestration of plant defences since their production is drastically enhanced when they are under biotic stress [49].

Biotic stress is responsible for significant sugarcane losses, posing a demand for the development of new stress-tolerant cultivars. In order to reduce insect and pathogen damage, plants have developed complex and varied defence mechanisms, including chemical and physical barriers. In the last few years, an extensive amount of work has been undertaken in order to decipher the sugarcane response to biotic stress, mainly related to some insect herbivores and pathogens. Amongst the SUCEST sequences, dozens of orthologous genes involved in the sugarcane response to insect herbivores [50] and *Diazothophic* endophytes [51, 52] were identified. Although the sugarcane-endophytic bacteria interaction is an advantageous association for both organisms, it is thought that sugarcane plants activate defence responses before the establishment of such symbiosis [53]. A study based on a wide gene expression analysis of 1,545 genes in sugarcane revealed that *Gluconacetobacter diazotrophicus* and *Herbaspirillum seropedicae* endophytic bacteria activated distinct classes of defence proteins, including four plant disease-resistant genes (R-genes), salicylic acid biosynthesis genes, five transcription factors, and so on. On the other hand, *Diatraea saccharalis* herbivory specifically upregulated the expression of a pathogenesis-related protein similar to thaumatin [37]. Transcript profiling of sugarcane-resistant plants to either *Ustilago scitaminea* or *Bipolaris sacchari* (also known as *Helminthosporium sacchari* or *Drechslera sacchari*), causal agents of smut and eyespot, respectively, identified 62 differentially regulated genes, of which 10 were downregulated and 52 were induced. Nineteen out of 52 transcript-derived fragments showed homology to known plant gene sequences, most being related to defense or signaling [54].

A considerable amount of data was obtained on how the plant hormone methyl jasmonate (MeJa) could be regulating plant defence reactions [28, 37, 55]. cDNA microarrays containing 829 ESTs from roots treated with MeJA, and 4793 ESTs from immature and mature stem tissues were used to evaluate gene expression changes produced by MeJA [28]. An MeJA solution was applied to the soil containing the plants, and the roots were harvested after 1, 3, and 10 days. The highest induction was observed for genes encoding the dirigentprotein, which is involved in lignin assembly and can protect plants against fungal attack [56]. Gene categories with increased transcript levels included signal transduction, the phenylpropanoid pathway, oxidative stress, and MeJA synthesis, indicating that several processes were altered by MeJA. In agreement with several studies involving transcription profiling, most of the up- or downregulated genes had unknown functions, reinforcing the great challenge of understanding plant gene function. Responses of sugarcane leaves sprayed with MeJA for 0.5, 1, 3, 6, and 12 hours, were investigated using nylon cDNA arrays containing 1536 ESTs from several cDNA libraries [55]. A total of 15 genes were upregulated, while 11 were downregulated. As observed in sugarcane roots [28], MeJA changed the expression of genes involved in several biological processes including transcription (a zinc finger protein), signalling (a protein kinase), and abiotic stress responses (a carboxy-peptidase, a peroxidase, and a heat shock factor). The authors complemented their analysis using a digital mRNA expression profiling of the differentially expressed genes, providing an overview of their expression patterns in different sugarcane tissues. These results support the idea that different in silicostrategies can be used to enrich functional genomics analyses.

Changes in gene expression in leaves exposed to MeJA were also evaluated using cDNA microarrays containing 1545 genes [37], mostly corresponding to signal transduction components [57]. The upregulation of transcription factors (MYB, NAC, and Aux/IAA) and histone homologues (H4 and H2B) strongly suggested chromatin remodelling followed by the activation of a cascade of signalling genes. Several protein kinases were up- and downregulated, indicating a complex network of sugarcane responses to MeJA.

Several strategies have been used to improve plant defence against insects and pathogens. The activation of stress-response transcription factors was found to enhance plant tolerance to fungal and bacterial pathogens in transgenic plants [58]. However, little is known about the function of other components of the plant transcription machinery during stress. The identification and characterization of agronomically-interesting genes related to herbivores and pathogens is a major challenge for sugarcane functional genomics. Several candidates have been tested in the last few years and incorporated into elite genotypes [59–66]. The heterologous expression of defence-related proteins in sugarcane, such as the soybean proteinase inhibitors encoding genes [67] or cry proteins from *Bacillus thuringiensis* [68], has led to increased resistance against the sugarcane borer *D. saccharalis*, the major sugarcane pest in Brazil. In addition, the molecular and functional characterization of cysteine proteinase inhibitors opened up new perspectives on pathogen control, since sugarcane cystatins inhibited the growth of the filamentous fungus *Trichoderma reesei*, suggesting that it can also be employed to inhibit the growth of pathogenic sugarcane fungi [69]. The use of inducible promoters will have a significant impact on the effectiveness and management of transgenic plants. One such promoter has been cloned in sugarcane that responds to the sugarcane borer (Silva-Filho, unpublished results). Taken together, the combination of new genes with appropriate regulatory sequences will be a major outcome of the sugar cane OMICS in breeding programs.

## 5. ASSESSING SUGARCANE GENES RELATED TO ABIOTIC STRESS

Plants face several restrictions in their environment and have developed a wide array of strategies to either avoid or cope with the stress condition. Most of the studies using high throughput assays, such as cDNA microarrays have been conducted with model plants, such as *Arabidopsis* or species not considered as tropical crops. Recently, the first insights into the responses of sugarcane to environmental stress have been provided.

Amongst abiotic stress, water deficit plays a major role, and increasing water scarcity has been observed throughout the world. Plant irrigation currently accounts for approximately 65% of global freshwater use, indicating that the development of drought-resistant plant varieties will be a necessity in the near future [70, 71]. Agricultural irrigation is one of the most water demanding human activities. In the case of sugarcane, agricultural frontiers are expanding, in part, in areas where irrigation is needed [72, 73].

To increase the knowledge on the sugarcane responses to drought, cDNA microarrays were used to evaluate gene expression in plants submitted to 24, 72, and 120 hours of water deprivation [37]. Drought stress caused dramatic changes in the gene expression profile of sugarcane plants, with 93 genes being up- or downregulated. Among the genes differentially expressed, transcription factor orthologs of the Myb, WRKY, NAC, and DREB proteins, which are known as role players in the drought responses of other systems [74–77], were upregulated. Sugarcane plants also selectively activated proteases in response to hydric stress, since a homologue to the cysteine proteinase RD19A precursor was induced. This gene is also induced by water stress in *Arabidopsis* [78].

Although it may sound surprising, another important stress in the case of sugarcane is cold stress, caused by temperatures below 0°C (freezing) or by low temperatures above 0°C (chilling). Cold stress is unusual in tropical areas, where most of the world's sugarcane is grown, but occasionally cold can severely affect crops in these regions. This is because most plants in the tropics have not developed strategies to avoid the devastating consequences of cold to the cells [79]. There is evidence that sugarcane varieties differ in their sensitivity to cold [80], suggesting the presence of alleles that might help this tropical crop to cope with this stress. These genes would have a great potential in breeding programs and also in the engineering of sugarcane plants with higher cold tolerance, a highly valuable trait that would allow the cultivation of this plant in temperate climates.

The first report of the use of cDNA arrays to discover sugarcane genes modulated by cold stress was conducted by Nogueira et al. [81]. The exposure of sugarcane plantlets to 4°C repressed the expression of 25 genes, while a further 34 genes were upregulated. Sugarcane homologues to several genes known to be induced by cold stress were found together with genes induced by drought in other species. This is probably because the cold induces the formation of ice, dehydrating the cell. Interestingly, 20 genes that had not previously been associated with cold or drought stress were identified, suggesting that sugarcane might activate novel cold response pathways. One example is the gene encoding a putative NAD-dependent dehydrogenase that might be involved in the protection against oxidative stress due to cold exposure. One of the genes, *SsNAC23*, is a member of the NAC family of transcriptional factors that are involved in biotic and abiotic stress and development [82]. In a further characterization of SsNAC23, Nogueira et al. [81] showed that the protein is targeted to the nucleus. In addition, *SsNAC23* transcripts also increased in response to herbivory and water stress. This data further reinforces the view that different kinds of stress may have common signalling pathways. Based on this expression profiling experiment, the authors proposed a hypothetical model integrating the several components activated by sugarcane in response to low temperature. The same data analyzed using PmmA [83] revealed a new set of 30 genes as differentially expressed. Among the genes upregulated was a putative endonuclease involved in nucleic acid repair, indicating that low temperature stress might cause DNA damage. Most genes in this new set were repressed by cold stress, such as those encoding a myo-inositol 1-phosphate synthase and an MAP Kinase.

Several plant responses to environmental stress are mediated by phytohormones, with a well-known cross-talk between them [84, 85]. To assess the role of ABA in sugarcane, Rocha et al. [37] sprayed ABA on sugarcane leaves and evaluated the gene expression profile using the cDNA arrays described above. Two genes encoding orthologs to receptor Ser/Thr kinases were upregulated. A phosphatase and a small GTPase were also induced, while a protein kinase was repressed. These findings help to depict an overview of the network of ABA signal transduction in sugarcane. The cDNA array data pinpointed several aspects of the sugarcane metabolism that seem to have been changed in response to ABA. For example, changes in the fatty acid composition probably take place, since a fatty acid desaturase was induced, while transpiration would be decreased due to the action of a PP2C-like protein homologous to ABI1 and ABI2. Moreover, the work of Rocha et al. [37] also showed drought responses similar to those elicited by ABA. For example, two delta-12 oleate desaturases, an S-adenosylmethionine decarboxylase, and a PP2C-like protein phosphatase were induced by both ABA and drought.

The cross-talk between ABA and MeJA also become evident from the activation of two genes involved in salicylic acid and MeJA biosynthesis in the ABA-treated plants [37]. ABA treatment elicited an antagonistic response between the ABA and auxin pathways. A gene coding for a protein similar to the auxin responsive protein GH3 [86] was found to be repressed by ABA. Furthermore, a gene coding for a protein with a predicted auxin-repressed domain found in dormancy-associated and auxin-repressed proteins [87] was upregulated by this hormone. The cross-talk of other hormone signalling pathways during water stress was further highlighted by the differential expression of several genes encoding proteins involved in ethylene, gibberellin, salicylic acid biosynthesis, as well as other proteins involved in hormone perception and action. In the same line, several genes induced by drought stress were also observed in sugarcane plants exposed to MeJA, suggesting that this hormone might play a role in gene expression changes during water deficit in this crop. These genes are interesting tools in the engineering of plants aimed at increasing drought tolerance. In fact, transgenic tobacco and rice plants over expressing a DREB protein and an NAC protein, respectively, had improved performance in response to water scarcity [88, 89].

Last, but not least, a study on the evaluation of sugarcane responses to low P availability was also reported. Most of the world’s agriculture takes place in soils with low availability of P and other nutrients [90]. Phosphorus, a key nutrient for plant growth and development, is taken up as inorganic phosphate (Pi), and most soils have very low Pi concentrations (around 2 mM) compared to the range 5–20 mM found inside the plant cells [91]. Soil supplementation with rock phosphate is widely used to increase P availability, increasing the productivity of several crops, including sugarcane [92]. However, since the P fertilizers may be exhausted within the next 60–90 years, and the P released into watercourses increases eutrophication of the water sources, there is a need to minimize P fertilization.

Phosphorus starvation experiments were used to access the changes in the gene expression profiles and gain information on the strategies used by sugarcane to overcome this nutrient deficiency stress [37]. The effect of P deficiency on the gene expression was evaluated in the roots of sugarcane plantlets. Fourteen genes were found to be repressed after 6 hours and 48 hours due to the absence of P in the nutrient solution. Surprisingly, no upregulated genes were identified. This was probably because of the highly stringent statistical test used, based on the outliers searching method [93]. When an alternative approach was used, based on the SOM algorithm [94], 146 genes were found, of which several were upregulated due to P stress [37]. This is an example of how the use of multiple statistical tests might improve the reach of large-scale gene expression profiling. Based on this larger set of genes, it was clear that P starvation triggered oxidative stress, since genes involved in the detoxification of reactive oxygen species, such as those encoding a gluthatione S-transferase and a superoxide dismutase, were induced. The role of GTPases in sugarcane responses to low P was pointed out by the differential expression of several small GTPases and their regulators, one Ran GTPase activator, one Rho GTPase activator, and one Rho GDP dissociation inhibitor. P starvation repressed one homologue of an auxin-repressed protein. The authors [37] found an interesting link between this protein and the fact that P-starvation in *Arabidopsis* caused an increase in the number of lateral roots, which is linked to increased auxin sensitivity. Interestingly, MeJA treatment also repressed the expression of this sugarcane gene, again showing a complex cross-talk between the hormones. Another indication of the hormonal regulation of the root architecture in response to low P levels was the repression of an EIL transcription factor, which was involved in root development in rice. The wide array of genes induced by P stress in sugarcane were in line with the complex responses observed in other species, such as tomato [95], *Arabidopsis* [96], white lupin [97], and rice [98].

## 6. THE SEARCH FOR REGULATORS OF SUGAR SYNTHESIS, TRANSPORT, AND ACCUMULATION

CO_2_ fixed during photosynthesis is used to synthesize carbohydrates [1, 99, 100]. Several adaptations were developed by some grass species, such as sugarcane and maize, aiming at optimizing CO_2_ fixation for carbohydrate biosynthesis. They developed a distinct carbon cycle, which defines them as the “C4 plants” [101]. The compound transported in bundle sheath cells (malate or aspartate), or the compound returned to the mesophyll cells (alanine or pyruvate), varies between the species. Also, different enzymes are involved in the decarboxylation reactions: phosphoenolpyruvate carboxykinase, NAD malic enzyme, and NADP malic enzyme (which is the case with sugarcane) [102]. In sugarcane leaves, CO_2_ fixation starts in the mesophyll cells, where CO_2_ is combined with phosphoenol pyruvate acid in a reaction catalysed by phosphenolpyruvate carboxylase. The resulting C4 compound, oxaloacetate, is converted to malate by NADP-malate dehydrogenase. Malate is transported to the bundle sheath cells and decarboxylated by the NADP-malic enzyme, releasing (and concentrating) CO_2_ for the RuBisCo action, which catalyzes the carboxylation of CO_2_ with ribulose-1,5-bisphosphate, the first step in the Benson-Calvin cycle. Glyceraldehyde 3-phosphate is formed and after several steps during which fructose-1,6-bisphosphatase (FBPase) and sucrose-phosphate synthase (SPS) play a major control role, sucrose is synthesized. Sucrose transfer to the phloem cells allows for its transport to the parenchyma cells located in the stem, the major sink tissue in sugarcane [100]. All these steps raise the possibility that the sugarcane sucrose content in the stem could be even higher, considering that it is possible, at least theoretically, to have higher rates of phloem loading transport to the stalks, to its parenchyma cells, and finally to the vacuoles of these cells, as well as the control of the use of sucrose for vegetative growth [100].

The interest in sugar transporters is obvious in sugarcane, and recent findings have indicated that sink strength is a driver for photosynthesis [103], highlighting their potential for sugarcane improvement. The SUCEST database contains nine monosaccharide and four disaccharide transporters (M. Menossi, unpublished results), and this diversity of transporters is in line with the findings that sugar transport involves either symplastic or apoplastic steps [104, 105]. An EST survey comparing transcripts from immature and mature internodes revealed transcripts encoding proteins homologous with known sugar transporters more abundant in the mature internodes [24]. The only sugarcane transporter showing high selectivity for sucrose, ShSUT1, was characterized in [106, 107]. This protein is supposed to act in the loading of sucrose from the vascular tissue into the parenchyma cells from the stem.

The large-scale analysis of gene expression in a population segregated for brix was used to identify genes associated with sucrose content [108]. The plants analyzed derived from multiple crossings among *S. officinarum* and *S. spontaneum* genotypes, and from commercial varieties selected for sugar content over 12–15 years. Sucrose accumulating internodes from field grown plants were assayed using cDNA microarrays containing 1545 elements. Transcriptome comparisons aimed at identifying differentially expressed genes were made by comparing high-sugar and low-sugar plants directly, and also by comparing high-sugar and low-sugar internodes. A total of 125 genes were found to have expression patterns correlated with sugar content. Genes encoding SNF-related kinases and involved in auxin signalling were found, providing insights into the regulatory network that might control sucrose accumulation. Intriguingly, several proteins related to stress responses, such as cytochrome P450 monoxygenases, were also found. Approximately half of the sucrose content-associated genes were found to be developmentally regulated during culm maturation, and many were related to stress responses. A comparison of this differential expression dataset with the results obtained when the plants were submitted to drought [37] revealed that approximately half of the genes identified as associated with the sucrose content were responsive to drought. They belonged to several functional categories including calcium signalling, stress responses, and protein phosphorylation. The data indicated that the sucrose accumulating tissues activate pathways during culm development, which overlap with drought and other stress responses such as cold and injury. This is corroborated by the observation that several SnRKs associated with the sucrose content and with drought belonged to the SnRK2 and SnRK3 family of kinases involved in osmotic stress responses [109].

The usefulness of evaluating progenies for gene expression studies has been reviewed by Casu et al. [110]. From their studies of a progeny contrasting for sucrose content, they showed that few of the differentially expressed genes were involved in carbohydrate metabolism. Additionally, a collection of 7409 ESTs from maturing sugarcane stems in combination with a smaller collection (1089) of ESTs from immature stems [23, 24, 111] were analyzed by bioinformatic techniques and by cDNA microarray methods, allowing for the identification of genes that are differentially regulated by stem maturity. The low level of sucrose metabolism gene expression observed indicated that when the culm matured and the sugar content increased, so sucrose synthesis and catalysis decreased. GeneChips from Affymetrix containing approximately 6,024 distinct *S. officinarum* genes were also used to study culm maturation, leading to the identification of the developmentally regulated genes involved in cellulose synthesis, cell wall metabolism, and lignification [112].

Source tissues might affect the efficiency and control of carbon fixation and allocation [113]. Gene expression in source tissues has been investigated in sugarcane using the EST analysis conducted by Ma et al. [27], and more recently using SAGE [114]. As pointed out before, the sugarcane photosynthetic carbon cycle is suspected to rely on the NADP malic enzyme pathway [115], but a high expression of a phosphoenolpyruvate carboxykinase was found in the bundle sheath cells from maize leaves, a species considered to employ the same mechanism as sugarcane [116, 117]. Recent evidence [114] indicated that the phosphoenolpyruvate carboxykinase might even be more active than the NADP malic enzyme in sugarcane leaves. This data highlighted how large-scale gene expression profiling can help in understanding complex traits such as sucrose content. In another study of sugarcane leaves, 24 genes were found to be differentially expressed in plants with high- and low-sugar content, selected from an F1 segregating population derived from a cross between two commercial sugarcane varieties [118]. Evidence that hormone signalling is related to the sucrose content was also found. One of the upregulated genes encoded an omega-3 fatty acid desaturase that might be involved in methyl jasmonate signalling. Other genes associated with high brix include a receptor-like serine/threonine kinase and a transcription factor containing an Myb domain. Surprisingly, from the 24 differentially expressed genes, 19 were more expressed in plants containing low-sugar content. Three of these genes encoded 14-3-3 like proteins, which have been found to reduce SPS activity [119, 120]. Another encoded an SNF1-related protein similar to a protein quinase that phosphorylates SPS in vitro [121] making it a target for the interaction with 14-3-3 proteins, which in turn reduces SPS activity. This data reinforced the usefulness of genomic approaches to uncover how sucrose metabolism can be regulated in sugarcane.

## 7. CONCLUDING REMARKS

Sugarcane cultivars tolerant to drought, cold, and poor soils are increasingly important in countries that are aiming to expand their plantations. The impending need to decrease fossil fuel usage together with the fact that ethanol is a less-pollutant renewable source of energy has renewed interest in the cultivation of sugarcane around the world, and many countries are developing an ethanol/biofuel industry. Cultivars adapted to grow in colder climates and high altitudes would be a highly attractive option. In Brazil, the largest sugarcane producer, sugarcane cultivation increased 11.20% in 2007 and the area planted increased by 7.4% [73]. This expansion occurred mostly in pastures and was possible due to the increased usage of irrigation and of new varieties adapted to the climate and to the soil of the regions. Small increases in sucrose content also contributed to the increased productivity.

Knowledge of the plant responses to drought, cold, and low levels of P help to provide a framework for improving sugarcane production using biotechnological tools. The possibility of using these genes as markers for breeding purposes or by genetic engineering of the sugarcane will certainly reduce the impact of the sugarcane crop on the environment. For example, the use of plants capable of growing in low-P soil would lead to reduced liming in the *cerrados* (savannas), which is a new agricultural frontier, and is characterized by low P availability. Additionally, as stated before, a large amount of the water used by men goes into agriculture, and improvements in sugarcane drought tolerance would reduce the impact on the water supply. A better understanding of how sugarcane plants cope with cold and drought stresses could aid in the development of cultivars better suited to particular areas.

The classical breeding of sugarcane takes 15 years of crosses and agronomical evaluation before a new cultivar is released for commercialisation. Gene discovery through the SUCEST sequencing program has been a major breakthrough for the breeding programs throughout the world, and functional studies based on cDNA arrays are uncovering pathways of plant adaptation and responses to the environment. EST-simple sequence repeats (SSRs) have been successfully used for genetic relationship analysis, extending the knowledge of the genetic diversity of sugarcane to a functional level. Development of new markers based on ESTs and their integration in genetic maps will renew breeding programs and help MAB technology speed up the breeding programs.

For over ten years now [122, 123] the directed genetic modification of sugarcane has been a reality in laboratories, and field trials have been conducted [124–127]. Genes can be silenced or overexpressed to study their function and to produce new phenotypes not possible through conventional breeding. Metabolic profiling associated with gene expression studies are certainly the future tools of the sugarcane industry. Also, the analysis of the transcriptome in transgenic plants altered for genes of interest would certainly prove to be an excellent tool to unravel sugarcane regulatory networks associated with important traits.
